# The Distribution of Li Ions in the Oxide Film Formed on Zircaloy-4 Corroded in Lithiated Water at 633 K

**DOI:** 10.3390/ma13040873

**Published:** 2020-02-15

**Authors:** Shijing Xie, Bangxin Zhou, Xue Liang, Qiang Li, Wenqing Liu, Meiyi Yao, Jinlong Zhang

**Affiliations:** Institute of materials, Shanghai University, Shanghai 200072, China; maxjing126@126.com (S.X.); liangxue@shu.edu.cn (X.L.); liqiang@shu.edu.cn (Q.L.); wqliu@staff.shu.edu.cn (W.L.); yaomeiyi@shu.edu.cn (M.Y.); jlzhang@shu.edu.cn (J.Z.)

**Keywords:** Zircaloy-4, corrosion resistance, Li ion segregation, oxide grain boundary, atom probe tomography

## Abstract

Transmission electron microscopy (TEM), second ion mass spectrum (SIMS) and atom probe tomography (APT) techniques are used to study the Li ion distribution in the oxide formed on the rolling surface (S_N_) of Zircaloy-4 corroded in lithiated water with 0.01 M LiOH at 633 K/18.6 MPa. The results showed that the Li ions segregated in the grain boundaries and subgrain boundaries in the oxide film, but nearly no Li ions were found in the oxide around the interface between the oxide and matrix. Finally, we discussed the mechanism of the LiOH influence on the corrosion resistance of Zircaloy-4.

## 1. Introduction

In pressurized water reactors (PWRs), zirconium alloys are widely used as fuel cladding materials. During working, LiOH is added in the coolant to adjust the pH value for reducing the corrosion rate of structure materials due to the addition of boric acid, in which the ^10^B acts as a burnable poison, but it is detrimental to the corrosion resistance of zirconium alloys. Therefore, a lot of research work has been performed in order to provide a better explanation of the mechanism concerning the degradation of corrosion resistance of zirconium alloys corroded in lithiated water.

Up to now, several mechanisms regarding the effect of lithiated water on the corrosion resistance of zirconium alloys have been proposed. For example, Cox et al. [1,2,3] suggested that m-ZrO_2_ or t-ZrO_2_ were present prior to dissolution in LiOH solution and where many pores formed in the oxide film, then the corrosion rate of zirconium alloys in lithiated water was accelerated. However, Liu et al. [4] raised a query where the driving force for the transport of dissolved ZrO_2_ to the solution came from. Jeong et al. [5,6] suggested that more vacancies could be formed in the oxide film when the Li^+^ substituted to Zr^4+^ so that it could enhance the corrosion rate. However, it is well known that the energy for the process of Li^+^ to replace Zr^4+^ in the oxide film is high; there is no way of knowing where the energy comes from. Pêcheur et al. [7,8] considered that the dense oxide layer could be modified and eliminated by Li ions and that this could be the reason for the enhancement of the corrosion rate of Zircaloy-4 corroded in lithiated water. Kim et al. [9] suggested that the Li ions can accelerate t-ZrO_2_ transformation to m-ZrO_2_ since the Li^+^ can substitute the Zr^4+^ in the oxide lattice; however, it supports the view proposed by Jeong [5,6]. In 2000, Pêcheur et al. [8] found that the concentration of Li ions at the interface between the oxide and matrix (O/M) dropped sharply, and they suggested that the Li ions could not enter in the dense oxide film. Thus, we can infer that the effect of Li ions on the transformation of t-ZrO_2_ to m-ZrO_2_ is limited. Ramasubramanian et al. [10] believed that the OLi groups could form on the oxide film surface, and could modify the normal oxide growth to accelerate the corrosion rate of zirconium alloys. However, up to now, there have been no reported results showing this kind of substance. Zhou et al. [11,12] held that the surface free energy of zirconium oxide could be decreased when Li ions were incorporated in the oxide. Therefore, the formation of defects in the oxide film could be enhanced, and the corrosion resistance was deteriorated.

Based on the discussion above, there are many possible mechanisms for the Li ions to influence the corrosion resistance of zirconium alloys, but further work is required. However, the mechanism of the effect of Li ions on the surface energy of oxide holds potential for future research. Although many articles about how LiOH affects the corrosion resistance of zirconium alloys have been reported, none have shown the distribution of Li ions in the oxide film in detail. Therefore, this was studied by transmission electron microscopy (TEM), atom probe tomography (APT) and second ion mass spectrum (SIMS) in the present work. We hope our results will be helpful for understanding the effect of Li ions distributing in oxide film on the corrosion behavior of zirconium alloys corroded in lithiated water.

## 2. Experimental Section

We used plates of Zircaloy-4 (Zr-1.20 Sn-0.23 Fe-0.11 Cr, wt.%) specimens about 24 × 15 × 2 mm^3^ in size. The specimens were annealed at 853 K for 10 h. Prior to the corrosion tests, these specimens were pickled in a mixed solution of s 10% HF +45% HNO_3_ + 45% H_2_O (in volume fraction), and rinsed in cold tap water and boiling water. Then, these specimens were exposed to lithiated water with 0.01 M LiOH at 633 K/18.6 MPa in a static autoclave.

In the reactor, only the rolling surface (S_N_) of the cladding tube reacts with coolant, so we only focused our attention on the oxide that formed on the S_N_ surface. The thickness of the oxide film was near 2 µm after corroding for 70 d. Then, TEM, SIMS and APT methods were used for analyses. The SIMS analysis was conducted by Physical Electronics (PHI) Adept 1010 SIMS (ULVAC-PHI, INC., Chigasaki, Kanagawa, Japan); the O^2+^ voltage was 3 kV, and the initial current was 200 nA. The diameter of the analysis spot was 300 μm. Needle-shaped atom probe specimens perpendicular to the S_N_ surface of the specimen were made using a Helios NanoLab^TM^ 600i focus ion beam (FIB) workstation (FEI Company, Hillsborough, OR, USA). Some TEM thin foil specimens of the cross-section of oxide film were also fabricated by the FIB method to observe the morphology of the oxide grains by JEM-2100 transmission electron microscope (JEOL Ltd., Akishima, Tokyo, Japan). The APT analysis was conducted by IMAGO LEAP 4000XHR with a laser pulsing mode at a frequency 125 kHz and 300 pJ laser energy (CAMECA, Gennevilliers, Hauts-de-Seine, France). During testing, the temperature of the specimens was maintained at 80 K, and the pressure in the analysis chamber was approximately 1.3 × 10^−10^ mbar. About 8.6 million data points were collected during analysis. Then, IVAS 3.6.8 software (3.6.8, CAMECA, Gennevilliers, Hauts-de-Seine, France) was used to analyze the experimental data. During analysis, we used the default *k*-factor of 3.3 and the evaporation field of 20 V/nm to reconstitute the maps of atomic distribution for different elements.

## 3. Results and Discussion

**Figure 1:**
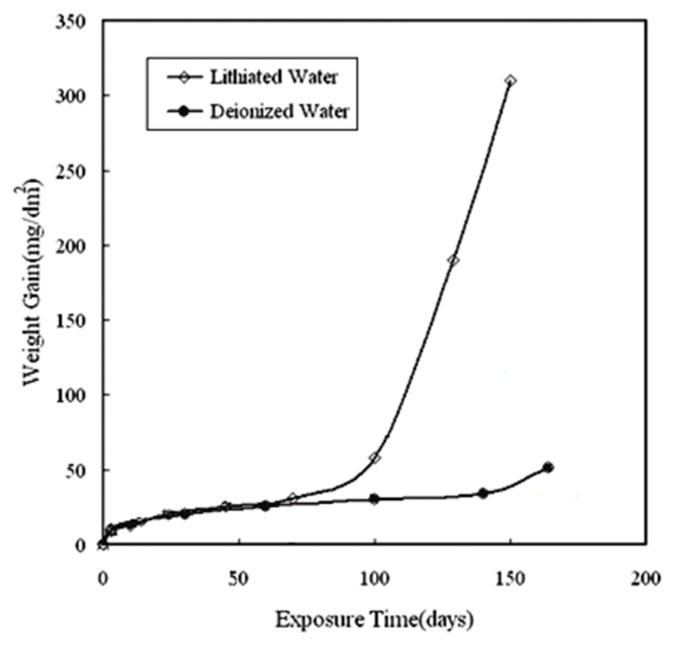
Weight gain versus exposure time for the Zircaloy-4 specimens corroded in lithiated water with 0.01 M LiOH and deionized water at 633 K/18.6 MPa.

Figure 1 shows the curve of weight gain versus exposure time for Zircaloy-4 specimens corroded in lithiated water with 0.01 M LiOH at 633 K/18.6 MPa. For comparison, the weight gain of Zircaloy-4 specimens corroded in deionized water is also depicted in Figure 1. After corroding for 100 d, the transition of the corrosion behavior of the specimens was obvious. Before 70 d exposure, the specimens were in the pretransition stage, and the weight gains of both specimens corroded in lithiated water and deionized water were nearly identical. After 70 d exposure, the thickness of the oxide film covered on the surface of the specimen corroded in lithiated water was about 2 µm (1 µm=15 mg/dm^2^ [13]). Geng et al. [14] evaluated the compressive stress in the oxide film (with the thickness between 0.5–2 µm), which covered the tubular specimens of Zircaloy-4 corroded in lithiated water and deionized water at 633 K/18.6 MPa after 3 to 100 d exposure by the “scrolled method” and the results showed that the compressive stress in the oxide layer for the specimens corroded in lithiated water was much lower than that corroded in deionized water. Their results showed that some influencing factors that affected the degradation of corrosion resistance of the specimens corroded in lithiated water had even existed in the oxide in the pretransition stage. Therefore, it is worthwhile to study the oxide which is formed in the pretransition stage at 70 d exposure to clarify the degradation mechanism of corrosion resistance for Zircaloy-4 corroded in lithiated water.

**Figure 2:**
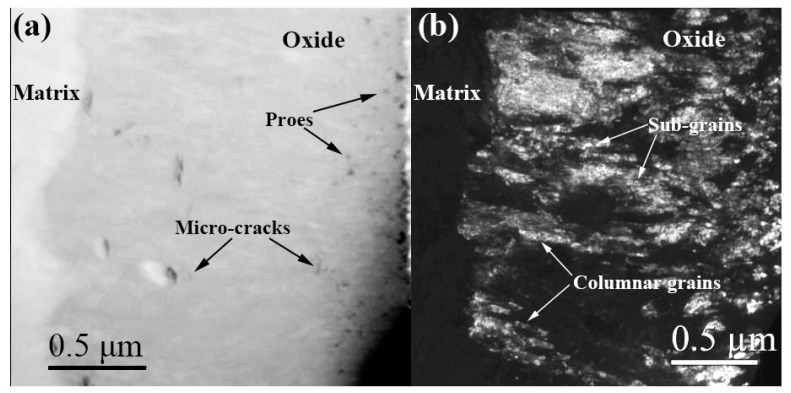
A high-angle annular dark-field (HAADF) image (**a**) and dark-field image (**b**) of the cross-section of the oxide film formed on the S_N_ surface of Zircaloy-4 corroded in lithiated water for 70 d.

Figure 2 is a high-angle annular dark-field (HAADF) image and dark-field image of the cross-section of the oxide film covered on the S_N_ surface of the specimen corroded in lithiated water after 70 d exposure. From Figure 2a, it is clearly shown that more pores and microcracks were distributed in the outer oxide film, but the inner oxide film was more dense. The length direction of the columnar grains was nearly perpendicular to the O/M interface, and many subgrains within the columnar grains can be clearly distinguished in Figure 2b.

**Figure 3:**
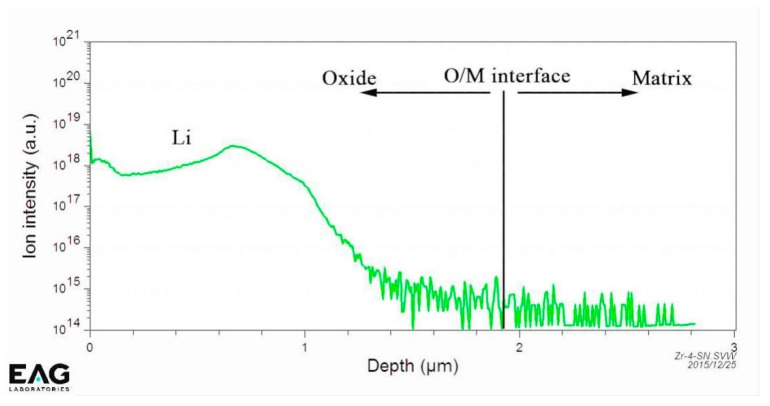
The distribution of Li^+^ across the thickness of oxide film formed on the S_N_ surface of the Zircaloy-4 specimen after corroding in lithiated water with 0.01 M LiOH at 633 K/18.6 MPa for 70 d.

Figure 3 shows the distribution of Li ions across the oxide film covered on the S_N_ surface of the Zircaloy-4 specimen, which was corroded in lithiated water with 0.01 M LiOH at 633 K/18.6 MPa for 70 d exposure, detected by SIMS. In Figure 3, the ordinate shows the ion intensity of Li^+^, and the abscissa represents the depth of oxide film. The depth at 0 means the outer surface of the oxide film, and the line marked on the abscissa at the depth near 2 μm identifies the O/M interface. In Figure 3, the ion intensity of Li^+^ is about 10^14^ in the O/M interface and matrix. However, the results reported by Jeong et al. [5] and Liu et al. [15] showed that there were nearly no Li ions distributed at the O/M interface and matrix. It seems that our results are contradictory to the reported results. In fact, this difference is attributed to there being no standard specimen calibrated in our test. Nevertheless, we consider that this result could have no effect on showing the difference of Li-ion distribution across the oxide film at different depths. Since no Li ions were distributed in the Zr matrix, we can judge that there was also no Li-ion distribution in the O/M interface based on the Li-ion concentration line in Figure 3, although the value of the Li-ion concentration was 10^14^ as shown in the ordinate. Therefore, we know the characteristics of the Li-ion distribution in the oxide film from Figure 3. The Li-ion concentration in the oxide film changed at different depths. On the outer surface of the oxide film, the Li-ion concentration was high since there are many pores and the oxide was looser [16]. With increasing oxide depth, the Li-ion concentration decreased, meaning that the oxide film in this part could be denser. However, the Li-ion concentration then increased with the increment of the oxide film depth and reached to another peak at about 0.7 μm in depth. The reason for this phenomenon could be that many defects in the oxide are distributed in this region. The Li-ion concentration then decreased rapidly with the increase in depth of the oxide film. The closer to the O/M interface, the denser was the oxide, so that there was nearly no Li-ion distribution around the O/M interface.

**Figure 4:**
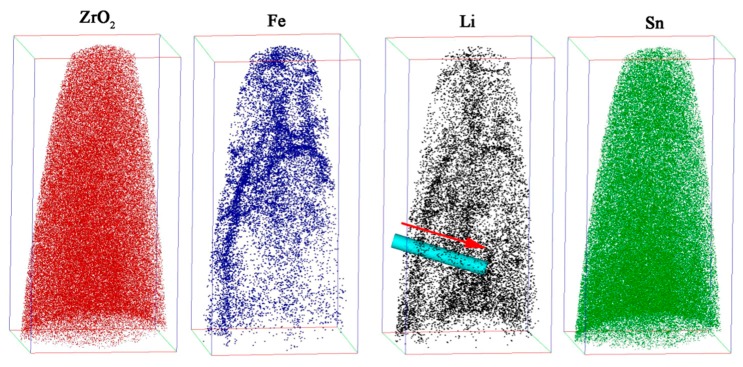
The distributions of ZrO_2_ (**red**), Fe (**blue**), Li (**black**) and Sn (**green**) in the needle-shaped specimen prepared in the oxide formed on the S_N_ surface of the Zircaloy-4 specimen corroded in lithiated water with 0.01 M LiOH at 633 K/18.6 MPa for 70 d exposure. The distance of the needle-shaped specimen tip apart from the O/M interface was about 1 μm. The size of the maps showing the atom distribution was 100 × 98 × 202 nm^3^. The analysis cylinder (10 nm in diameter) is signed in the graph, and the arrow indicates the analysis direction for Figure 5.

**Figure 5:**
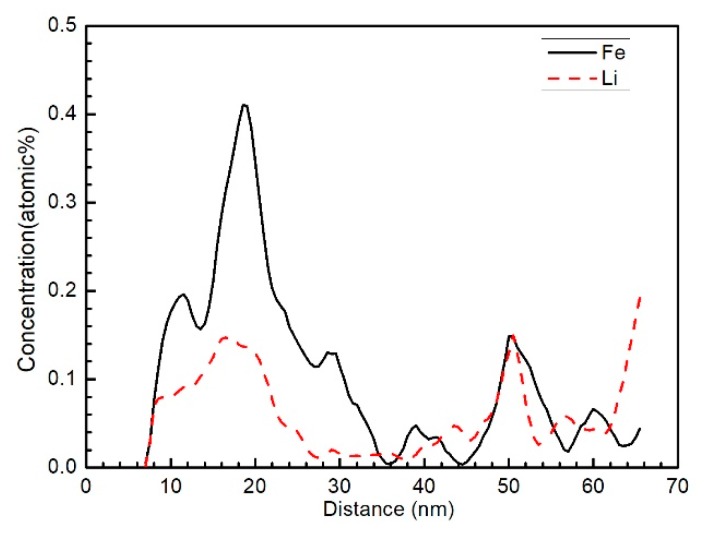
The concentration curves of Fe and Li in the analysis cylinder (as shown in Figure 4).

The 3D reconstruction maps for ZrO_2_ (red), Fe (blue), Li (black) and Sn (green) in the needle-shaped specimen prepared in the oxide film were about 1 μm apart from the O/M interface, as shown in Figure 4. The size of the maps showing the atom distribution was 100 × 98 × 202 nm^3^. It is clearly shown that the Fe atoms segregated in some regions of the oxide rather than in a uniform distribution. The distribution of Li-ions was also not uniform, and the Li-ions segregated in the positions similar to that of the Fe atoms. The Sn atoms were uniformly distributed in the oxide. The Cr atoms were not detected in the present test. When comparing the morphology shown in Figure 2 and Figure 4, particularly the 3D pattern of Fe and Li distribution, by rotating the atom distribution map, it is obvious that the segregation of Li ions and Fe atoms shown in Figure 4 was on the grain boundaries and subgrain boundaries of columnar oxide grains. In order to clearly show the Li-ion segregation at the oxide grain boundaries or subgrain boundaries, an analysis cylinder (10 nm in diameter shown in Figure 4) was used, perpendicular to the oxide grain boundary; the arrow indicates the analysis direction. The concentration curves of Fe and Li in the analysis cylinder are showed in Figure 5. It is clearly shown that Fe and Li segregate in the same regions. The distance of the concentration peaks of Fe and Li are nearly equal to the size of the subgrains in dozens of nanometers. There is no doubt that the Li ions segregated at the grain boundaries or subgrain boundaries of the oxide instead of being uniformly distributed in the oxide.

**Figure 6:**
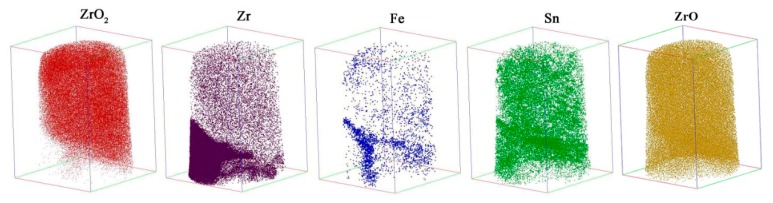
The distribution of ZrO_2_ (**red**), ZrO (**yellow**), Zr (**purple**), Fe (**blue**) and Sn (**green**) in the needle-shaped specimen prepared in the oxide covered on the S_N_ surface of the Zircaloy-4 specimen corroded in lithiated water with 0.01 M LiOH at 633 K/18.6 MPa for 70 d exposure. The specimen was around the O/M interface. The size of the maps showing the atom distribution was 73 × 71 × 98 nm^3^.

**Figure 7:**
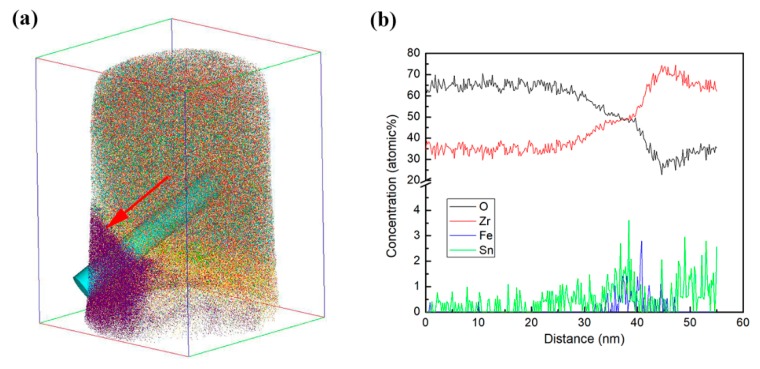
A 3D map of all the substances’ atoms, ZrO_2_ (red), ZrO (yellow), Zr (purple), Fe (blue) and Sn (green) in needle-shaped specimen prepared in the oxide covered on the S_N_ surface of the Zircaloy-4 specimen after corroding in lithiated water with 0.01 M LiOH at 633 K/18.6 MPa for 70 d. The specimen was around the O/M interface. The size of maps showing the atom distribution was 73 × 71 × 98 nm^3^. The analysis cylinder (10 nm in diameter) is signed in the graph, and the arrow indicates the analysis direction (**a**). The concentration curves of Zr, O, Fe and Sn in the analysis cylinder (**b**).

Figure 6 shows the 3D reconstruction maps for ZrO_2_ (red), ZrO (yellow), Zr (purple), Fe (blue) and Sn (green) obtained by another needle-shaped specimen fabricated around the O/M interface. ZrO_2_ was mainly located in the upper-right part of the map, and Zr was mainly located in the lower-left part of the map. The distributions of Fe and Sn were nonuniform, and there was Fe and Sn segregation in localized areas. Figure 7a is the 3D map of all the atoms; an analysis cylinder (10 nm in diameter) was used through the interface of the Zr and ZrO_2_ enrichment region. From the concentration curves of Zr, O, Fe and Sn in the analysis cylinder shown in Figure 7b, we know that an O/M interface was located in this specimen. From the combined distribution of Fe and Sn atoms in Figure 6, we can conclude that there was Sn and Fe segregation at the O/M interface, but no Li ions were detected on or around the O/M interface in the oxide, since no peak was located at 7 in the mass-to-charge state-ratio in mass spectrum that indicates Li during APT detection. The regular pattern of Li-ion distribution in the oxide film observed by APT, shown in Figure 4 and Figure 6, is in accord with the result obtained by SIMS analysis shown in Figure 3.

Based on the discussion in the introduction, we know that the mechanism of the Li ions’ effect on the surface energies of the grain boundaries of the oxide is more reasonable than others. So, we will discuss the mechanism of the LiOH influence on the corrosion resistance of Zircaloy-4 from this angle. The oxidation reaction between oxygen and zirconium to form ZrO_2_ occurs at the O/M interface. Therefore, the oxide grain boundaries provide an important path for the corrosion medium’s diffusion into the oxide film during the growth of the oxide [17,18]. If the characteristics of the grain boundaries are altered by some factors, the corrosion resistance of specimens should definitely be changed.

TEM and scanning electric microscope (SEM) techniques were employed to examine the microstructure and the fracture surface morphology of the oxide film covered on the Zircaloy-4 specimens corroded in lithiated water and deionized water at 633 K/18.6 MPa by Zhou et al. [11,12]. Their results showed that more pores and microcracks formed along the grain boundaries in the oxide film covered on the specimens corroded in lithiated water than that corroded in deionized water. The energies of the grain boundaries of ZrO_2_ formed on the Zircaloy-4 specimen corroded in lithiated water would be lower than that corroded in deionized water since the Li ions segregated in the grain boundaries. So, the gathering of defects in the grain boundaries of the ZrO_2_ formed on the Zircaloy-4 specimen corroded in lithiated water could have been easier than that corroded in deionized water. Scanning probe microscopy (SPM) was employed to examine the surface morphology of the oxide covered on the specimens corroded in lithiated water in the early stage of pretransition [11]. Based on the difference of the surface undulation of oxide grains, it was suggested that the surface free energy of the oxide formed on the specimen corroded in lithiated water was lower. When the surface free energy was lower, the tendency of the surface area to increase was greater. Therefore, the incorporation of Li ions into the oxide can promote the formation of pores and microcracks by the condensation of vacancies due to the reduction of surface free energy of the oxide as it was proposed in References [11,12]. However, there was no clear picture to show where Li ions were located by the experimental results at that time. In this study, the distribution of Li ions on the grain boundaries and subgrain boundaries of the oxide in the pretransition stage of corrosion was clearly revealed by APT analysis. For this reason, the compressive stress in the oxide film was effectively released as the defect formation was prompted by Li ions. Thus, we can understand the phenomena that the compressive stress was lower in the oxide film covered on the surface of specimens corroded in lithiated water than that corroded in deionized water even in the pretransition stage of the corrosion process, which was reported by Geng et al. [14]. Then, more defects distributed along the grain boundaries in the oxide film covered on the surface of specimens corroded in lithiated water than that corroded in deionized water after corrosion transition [11,12]. It is easy to understand why the same specimen corroded in lithiated water leads to a shorter transition period and faster corrosion rate after corrosion transition than that in deionized water, as shown in Figure 1.

The findings of this study help to better understand the promotion of the enlargement or extension of the pores in the oxide film formed on the specimens corroded in lithiated water, as suggested by Cox et al. [1,2,3], and also the Li ions could modify the characteristics of the inner barrier layer of the oxide film to prompt the disappearance of the dense barrier layer, proposed by Pêcheur et al. [7,8], can also be better understood in light of such a pattern of Li ion distribution in oxide.

## 4. Summary

The distribution of Li ions in the oxide film formed on the Zircaloy-4 specimen, which was corroded in lithiated water with 0.01 M LiOH at 633 K/18.6 MPa for 70 d exposure was investigated by TEM, SIMS and APT methods. The results showed that the Li ions segregated in the grain boundaries and subgrain boundaries of the oxide rather than as a uniform distribution in the oxide film, but nearly no Li ions were detected at or around the O/M interface of the specimen. It was suggested that the degradation mechanism for the corrosion resistance of the Zircaloy-4 specimens corroded in lithiated water was attributed to the promotion of the formation of pores and microcracks along the oxide grain boundaries and subgrain boundaries, due to the segregation of Li ions there.
